# Phase-controlled van der Waals growth of wafer-scale 2D MoTe_2_ layers for integrated high-sensitivity broadband infrared photodetection

**DOI:** 10.1038/s41377-022-01047-5

**Published:** 2023-01-02

**Authors:** Di Wu, Chenguang Guo, Longhui Zeng, Xiaoyan Ren, Zhifeng Shi, Long Wen, Qin Chen, Meng Zhang, Xin Jian Li, Chong-Xin Shan, Jiansheng Jie

**Affiliations:** 1 School of Physics and Microelectronics, Key Laboratory of Material Physics Ministry of Education, Zhengzhou University, Zhengzhou, Henan 450052 China; 2 Department of Electrical and Computer Engineering, University of California San Diego, La Jolla, CA 92093 USA; 3 Institute of Nanophotonics, Jinan University, Guangzhou, Guangdong 511443 China; 4 Institute of Functional Nano and Soft Materials (FUNSOM), Jiangsu Key Laboratory for Carbon-Based Functional Materials and Devices, Soochow University, Suzhou, Jiangsu 215123 China

**Keywords:** Photonic devices, Imaging and sensing

## Abstract

Being capable of sensing broadband infrared (IR) light is vitally important for wide-ranging applications from fundamental science to industrial purposes. Two-dimensional (2D) topological semimetals are being extensively explored for broadband IR detection due to their gapless electronic structure and the linear energy dispersion relation. However, the low charge separation efficiency, high noise level, and on-chip integration difficulty of these semimetals significantly hinder their further technological applications. Here, we demonstrate a facile thermal-assisted tellurization route for the van der Waals (vdW) growth of wafer-scale phase-controlled 2D MoTe_2_ layers. Importantly, the type-II Weyl semimetal 1T′-MoTe_2_ features a unique orthorhombic lattice structure with a broken inversion symmetry, which ensures efficient carrier transportation and thus reduces the carrier recombination. This characteristic is a key merit for the well-designed 1T′-MoTe_2_/Si vertical Schottky junction photodetector to achieve excellent performance with an ultrabroadband detection range of up to 10.6 µm and a large room temperature specific detectivity of over 10^8^ Jones in the mid-infrared (MIR) range. Moreover, the large-area synthesis of 2D MoTe_2_ layers enables the demonstration of high-resolution uncooled MIR imaging capability by using an integrated device array. This work provides a new approach to assembling uncooled IR photodetectors based on 2D materials.

## Abstract (web summary)

Wafer-scale phase-controlled 2D MoTe_2_ layers was synthesized for in-situ fabrication of room-temperature high-performance broadband infrared photodetector and device array.

## Graphical abstract

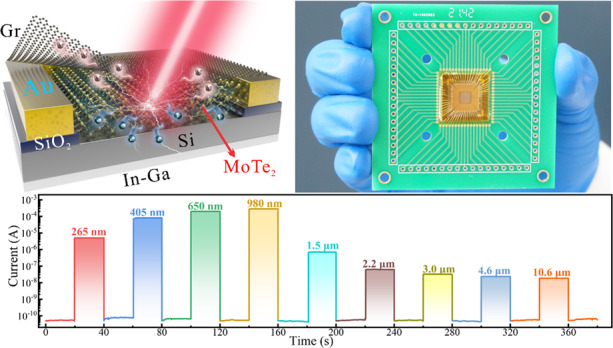

## Introduction

Detection in multiple infrared (IR) regions spanning from short- and mid- to long-wave IR plays an important role in diverse fields, from scientific research to wide-ranging technological applications, including target identification, imaging, remote monitoring, and gas sensing^1–3^. Currently, the state-of-the-art IR photodetectors are mainly dominated by conventional narrow bandgap semiconductors, including In_1-x_Ga_x_As, InSb, and Hg_1-x_Cd_x_Te, operating in short-wave IR (SWIR, 1–3 µm), mid-wave IR (MWIR, 3–6 µm), and long-wave IR (LWIR, 6–15 µm) spectral bands, respectively^4–6^. Notably, these photodetectors not only rely on expensive processes of raw materials growth and complex processing procedures, but also suffer from cryogenic cooling conditions with time-consuming and high power consumption^7,8^. Moreover, there are several remaining technological challenges, such as poor complementary metal-oxide-semiconductor (CMOS) compatibility, bulky module size, and low efficiency, which severely restrict the wider application of these detectors. Additionally, similar limitations and drawbacks associated with other mature semiconductor technologies, such as quantum wells, type-II superlattices, and III-V semiconductor heterostructures, have stimulated the research community to search for new IR absorber candidates, which not only possess good optoelectronic properties comparable to III-V material systems but also feature low-cost, homogenous integration, ease of fabrication, high-yield production^4^. To date, several alternative candidates, e.g., colloidal quantum dots (CQDs), graphene, black phosphorus (BP), and black arsenic phosphorus (b-AsP), have been intensively studied^9–14^. These promising alternatives have enabled advanced achievements in IR photodetection to overcome classical counterparts’ limitations. For instance, room temperature SWIR detection (λ = 1.3 µm) has been demonstrated based on solution-processed PbS quantum dots (QDs)^15^. MWIR-LWIR detection has been realized with two-dimensional (2D) material systems of BP and b-AsP (3–8 µm), at room temperature^16–18^. However, several unsolved challenging issues of these materials in terms of low optical absorption, chemical instability, non-scalable fabrication, and complicated manufacturing techniques impede their further technological actualization and commercialization^19,20^. Therefore, the exploration of an attractive and promising candidate with wide bandgap tunability, strong IR absorption, good air stability, and excellent optoelectronic properties may offer an alternative solution for high-sensitivity IR detection.

Recently, the rising star of 2D molybdenum ditelluride (MoTe_2_) with a favorable transition from semiconducting (2H) to semimetallic (1T′) phase, high carrier mobility, wide optical absorption, and good chemical stability has been chosen as a promising candidate for the IR sensors assembly^21–24^. Compared with its semiconducting counterpart with a bandgap of ~1.0 eV, semimetallic 1T′-MoTe_2_ featuring a gapless nature and unique optoelectronic properties, has shown great potential for the use in broadband IR photodetection applications. More importantly, 1T′-MoTe_2_ has been confirmed to be a type-II Weyl semimetal^22,23^, where the Weyl point appears at the boundary of electron and hole pockets, enabling the realization of a high sensitivity response over a wide spectral band due to the linear dispersion and suppressed backscattering^24^. To date, top-down exfoliation of MoTe_2_ from the bulk crystal was stuck by the low yield and limited size. Although bottom-up growth of MoTe_2_ could be achieved by the standard chemical vapor deposition (CVD) methods, the unsatisfactory lateral scale of resultant few or multilayer nanosheets is limited to tens of micrometers, and even smaller sizes^25^. So far, the demonstration of a number of broadband photodetectors utilizing micron-sized 2D semimetals in several studies suggests their initial potential in next-generation IR photodetection technologies^24^. Unfortunately, the main technological bottlenecks directly related to the large noise level under a bias voltage and the low charge separation efficiency impede their further applications in high-performance broadband IR photodetection due to the lack of high-quality junctions^26,27^. On the other hand, the weak optical absorption originating from atomically thin 2D materials results in another limitation of the total photocarrier generation in photodetectors^28^. Moreover, by applying a bias voltage on the metal-semimetal-metal device, the large dark current through the photodetector will significantly reduce the specific detectivity of the detector^26^. We, therefore, propose the construction of 2D/three-dimensional (3D) mixed-dimensional van der Waals (vdW) heterostructures to boost the performance of detectors, which can combine novel 2D materials and 3D semiconductors with mature integration processes^29,30^.

Herein, we propose a facile and scalable thermal-assisted tellurization approach for the rational growth of wafer-scale 2D MoTe_2_ layers with controllable thickness, along with a tunable phase transition from semiconductor (2H) to semimetal (1T′). The large-scale uniform 2D MoTe_2_ layers enable the in situ construction of a high-quality vertical 1T′-MoTe_2_/Si 2D/3D Schottky junction device. The as-fabricated photodetector is capable of sensing broadband IR light up to the LWIR range (λ = 10.6 µm) at room temperature with large specific detectivities of 4.75–2.8 × 10^8^ Jones in the mid-infrared (MIR) range of 3.0–10.6 µm, which is among the widest range for photodetectors based on 2D transition metal chalcogenides (TMDs) materials. Importantly, the nature of the thermal-assisted tellurization technique allows for the assembly of an 8 × 8 focal plane array device, demonstrating an impressive MIR imaging capability without the need for cryogenic cooling. This work provides a viable way for the development of highly sensitive 2D photodetectors for room temperature MIR photodetection and image-sensing applications.

## Results

**Fig. 1:**
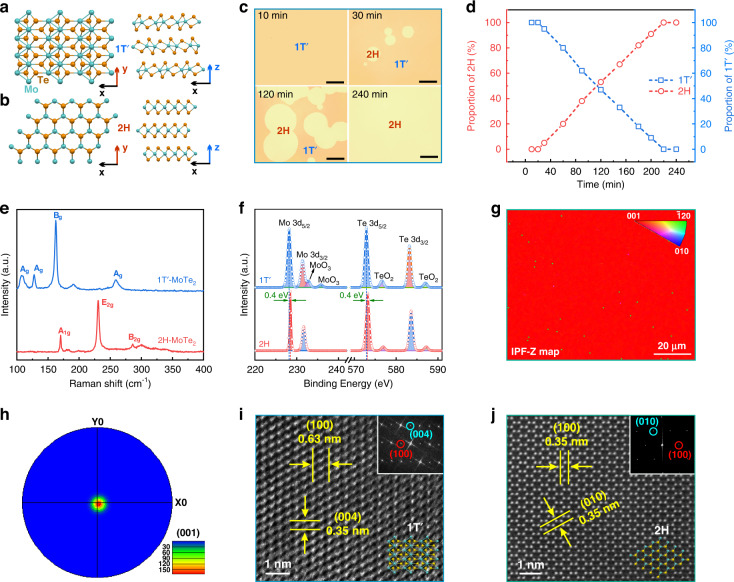
Synthesis and characterization of the 2D MoTe_2_ layers. **a**, **b** Atomic structures of 1 Tʹ- and 2H-MoTe_2_. **c** Photographs of the 2D MoTe_2_ layers with different growth times. Scale bar: 100 μm. **d** Proportion of 1 Tʹ- and 2H-MoTe_2_ as a function of growth time. **e**, **f** Raman and XPS spectra of the 1 Tʹ- and 2H-MoTe_2_ layers. **g**, **h** Inverse pole figures along the *z*-axis (IPF-Z) and pole figure of the 1Tʹ-MoTe_2_ layers acquired from EBSD mapping on large-area. **i, j** Z-contrast STEM images of 1Tʹ- and 2H-MoTe_2_

Figure 1a, b show the atomic structures of the semimetallic 1T′ (monoclinic) and semiconducting 2H (hexagonal) phases of MoTe_2_, respectively. For the 2H phase, a single MoTe_2_ layer consists of a Mo atoms layer arranged in hexagons between two Te atoms layers in repetitive cells. In contrast, Mo atoms arrange octahedral coordination around the Te atoms, with lattice distortion along the *x*-axis to form the 1T′ phase^31^. In this work, a pre-deposited Mo film as a precursor was transformed into 2D MoTe_2_ layer via a vdW growth mechanism through a direct thermal-assisted tellurization process, as shown in Fig. S1. As a matter of fact, the phase transition of MoTe_2_ is highly dependent on the growth time. Optical images in Fig. 1c reveal that the 1T′-MoTe_2_ layer was readily obtained by transforming the pre-deposited Mo metal layer within a short growth time of 10 min due to the decreased free energy of 1T′-MoTe_2_ in the presence of the Te deficiency in the initial transition stage^31^. Note that the transition time from Mo to 1T′-MoTe_2_ is highly dependent on the initial thickness of the Mo layer, which increases with the increasing precursor Mo layer thickness (Fig. S2). As the growth time increases to 30 min, the island-shaped 2H-MoTe_2_ layers only appear randomly, and then gradually become larger as the growth time increases to 120 min. Specially, when the growth time further reaches 240 min, the island-like layers of 2H-MoTe_2_ merge together, leading to the formation of a uniform and continuous 2H-MoTe_2_ layer. The phase transformation from 1T′ to 2H phase can be elucidated by the time-temperature-transformation (TTT) theory^32^. Figure 1d plots the proportion of 1Tʹ and 2H phases of MoTe_2_ with the growth time, revealing the linear increase of the proportion of 2H-MoTe_2_ layers with increasing the growth time at a rate of ~0.5% per min.

Based on the Raman spectroscopic studies of the as-synthesized 1Tʹ- and 2H-MoTe_2_ layers in Fig. 1e, the phase-specific characteristic Raman peaks of *B*_*g*_ (162.5 cm^−1^) and *A*_*g*_ (258.6 cm^−1^) vibration modes for 1T′-MoTe_2_ and *A*_*1g*_ (170.3 cm^−1^) and *E*_*2g*_ (230.5 cm^−1^) for 2H-MoTe_2_, respectively, can be clearly distinguished. In the X-ray photoelectron spectroscopy (XPS) analysis (Fig. 1f), the peaks of Mo 3d_5/2_ (228.5 eV), Mo 3d_3/2_ (231.7 eV), Te 3d_5/2_ (573.2 eV), and Te 3d_3/2_ (583.6 eV) can be assigned to 2H-MoTe_2_, while the corresponding peaks of 1 T′-MoTe_2_ redshift by ~0.4 eV compared to the semiconducting phase due to the reduction in binding energy caused by Te deficiency^33^. Besides, the atomic ratios of Te/Mo are evaluated to be ∼1.92 and ~1.98 for the 1Tʹ- and 2H-MoTe_2_ layers, respectively, confirming the existence of more Te deficiency in the 1Tʹ-MoTe_2_ layer. As shown in Fig. S3, the X-ray diffraction (XRD) patterns of both the 1Tʹ- and 2H-MoTe_2_ layers present four sharp peaks assigned to crystal planes of (002), (004), (006), and (008), respectively, without Mo peaks, indicating the full transformation of the Mo film to highly crystalline MoTe_2_ layers along the (001) direction in a preferential orientation. Transmission electron microscopy (TEM) results in Fig. S4 indicate that 2D MoTe_2_ layers consist of many adjacent single-crystal MoTe_2_ domains. Furthermore, electron back-scattered diffraction (EBSD) mapping conducted on the large-area MoTe_2_ sample with the uniform red color (related to the [001] crystal orientation) in Fig. 1g confirms the preferential crystal growth of the 1Tʹ-MoTe_2_ layers along the normal direction, consistent with the XRD results. In addition, a number of crystal domains arranged in a mosaic of a single crystal for layers of 1Tʹ-MoTe_2_ is further evidenced by the inverse pole figure maps along the *x*- and *y*-axes in Fig. S5. The (001) dominant orientation in the MoTe_2_ layers, as confirmed by the pole figure in Fig. 1h, demonstrates high crystallinity despite polycrystalline features. The Z-contrast high-resolution scanning TEM (HRSTEM) images in Fig. 1i, j show a monoclinic lattice structure with spacing distances of 0.35 and 0.63 nm, which correspond to (100) and (004) planes of 1Tʹ-MoTe_2_, while 2H-MoTe_2_ possesses a hexagonal atomic structure with a lattice spacing of 0.35 nm for the (100) and (010) planes.

**Fig. 2:**
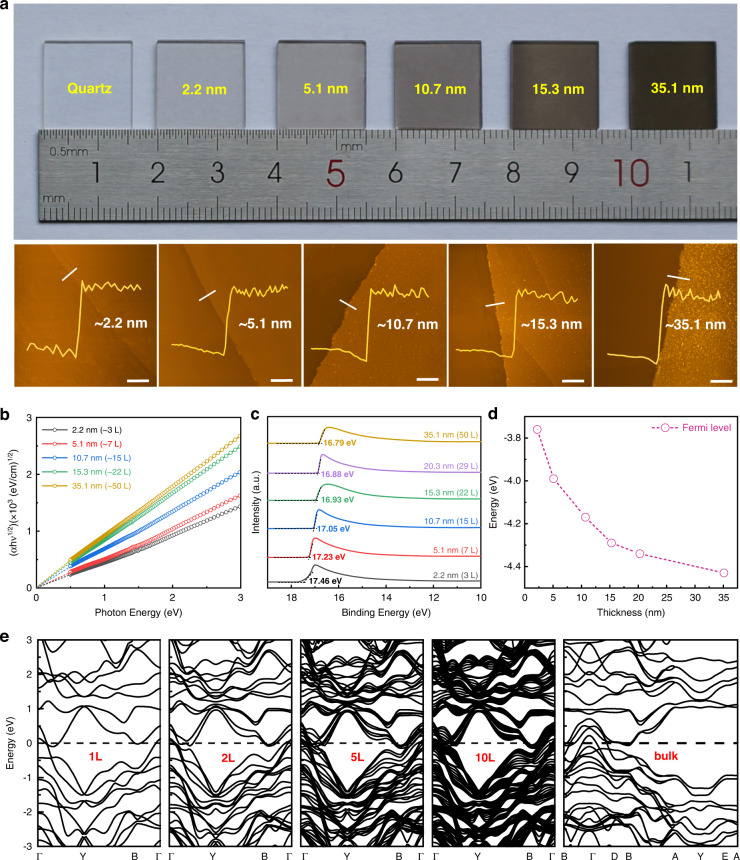
Thickness control and characterization of the 2D MoTe_2_ layers. **a** Photograph and corresponding AFM images of the 1Tʹ-MoTe_2_ layers with different layer numbers grown on the quartz. Scale bar: 2 μm. **b** Thickness-dependent Tauc plots of the 1Tʹ-MoTe_2_ layers. **c**, **d** Secondary-electron cut-off regions and Fermi levels of the 1Tʹ-MoTe_2_ layers. **e** Electronic band structures of the 1Tʹ-MoTe_2_ layers obtained from DFT simulation

By virtue of the facile and scalable thermal-assisted tellurization strategy, we can precisely tailor the thickness of the 2D MoTe_2_ layers by tuning the initial Mo film thickness. Figure 2a demonstrates the 2D 1Tʹ-MoTe_2_ layers with various thicknesses of ~2.2, ~5.1, ~10.7, ~15.3, and ~35.1 nm, according to atomic force microscopy (AFM) results. The thickness-dependent XRD patterns and Raman spectra of the 1Tʹ-MoTe_2_ layers in Fig. S6 confirm the high quality of as-synthesized samples with well-controlled thickness. From the measured absorption spectra in Fig. 2b, the layer-independent optical bandgap with zero bandgaps strongly manifests its gapless nature for 1Tʹ-MoTe_2_ layers. In Fig. 2c and Fig. S7, ultraviolet photoemission spectroscopy (UPS) characterization of 1Tʹ-MoTe_2_ with different layer thicknesses reveals the layer-dependent energy band structure. The work function (*W*_F_) values are calculated to be ~3.76, ~3.99, ~4.17, ~4.29, ~4.34, and ~4.43 eV for the 1Tʹ-MoTe_2_ layers with thicknesses of ~2.2, ~5.1, ~10.7, ~15.3, ~20.3, and ~35.1 nm, respectively. Note that the Fermi level (*E*_F_) shifts from 3.76 to 4.43 eV due to the higher hole concentration in the relatively thicker MoTe_2_ layers (Fig. S8), as plotted in Fig. 2d. In addition, the Hall measurements confirm that carrier mobility highly depends on layer thickness, revealing the increased mobility and decreased resistivity of 2H/1Tʹ-MoTe_2_ layers with increasing the layer thickness. Specifically, the 1Tʹ-MoTe_2_ layers possess a higher carrier mobility than their its 2H phase counterparts, which is beneficial for improving the carrier collection efficiency. To further verify the above experimental observation, we carried out density functional theory (DFT) calculations for simulating the layer-dependent electronic band structures of the 2D MoTe_2_ layers, as shown in Fig. 2e, from which the gapless nature of 1Tʹ-MoTe_2_ can be clearly observed from monolayer to bulk. In contrast, 2H-MoTe_2_ has an indirect bandgap of 1.16–0.73 eV for its monolayer to bulk structure (Fig. S9). In addition, the scalable and facile thermal-assisted tellurization route enables the direct growth of wafer-scale 2D MoTe_2_ layers on 2-inch SiO_2_/Si wafers, as shown in Fig. S10a, b. Besides, the Raman scans across the wafer diameter with similar density and profile in Fig. S10c, d suggest the admirable homogeneity and uniformity of the large-scale 2D MoTe_2_ layers, making them attractive and promising candidates for application in integrated optoelectronic devices and systems.

**Fig. 3:**
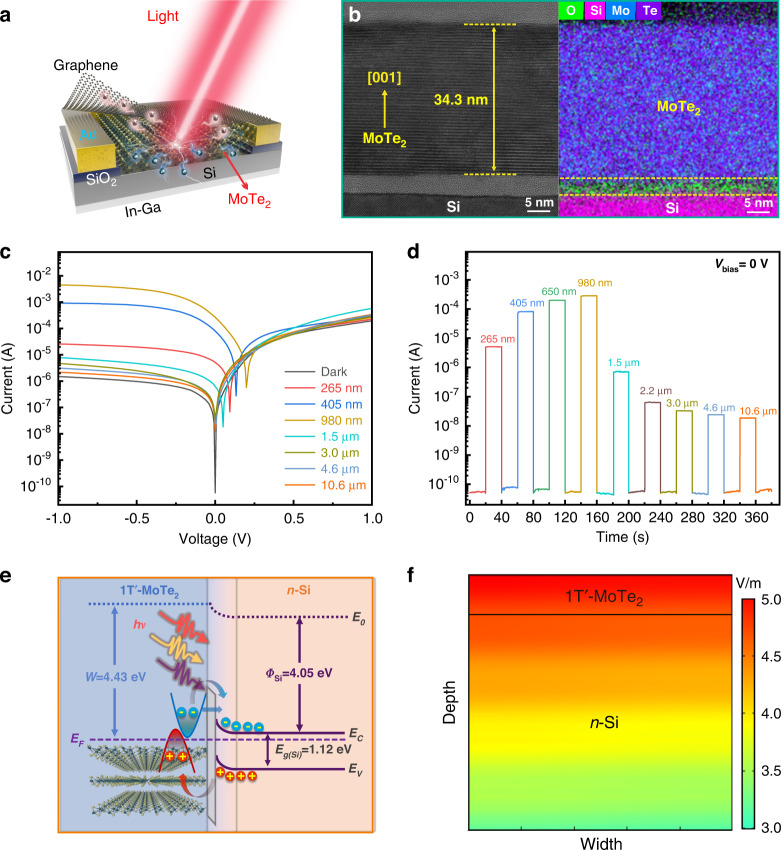
Optoelectronic performance of the photodetector. **a** Schematic illustration of a graphene/1Tʹ-MoTe_2_/Si Schottky junction device. **b** Cross-sectional HRTEM image of the device and the corresponding elemental mapping image. **c** *I-V* characteristics of the graphene/1Tʹ-MoTe_2_/Si Schottky junction device acquired in the dark and under irradiation of broadband light. **d** Time-dependent photoresponse properties to pulsed light illumination in a broad spectral band. **e**, **f** Energy band diagram and electrical potential distribution of the photodetector

The vdW growth of the large-area 2D MoTe_2_ layers offers more flexibility for the development of high-sensitivity optoelectrical devices. In light of this, we developed a 1Tʹ-MoTe_2_/Si vertical Schottky junction device by the in situ vdW growth of 1Tʹ-MoTe_2_ layers on a pre-patterned Si (*n*-type, 1–10 Ω cm) substrate. To ensure efficient carrier collection, monolayer graphene (Gr, 300 Ω sq^−1^) and In-Ga alloy were selected as top transparent contact with 1Tʹ-MoTe_2_ layer and an ohmic contact with Si (Fig. S11), respectively. Figure 3a shows the schematic illustration of the Gr/1Tʹ-MoTe_2_/Si Schottky junction device, and the photograph of a real device is shown in Fig. S12. The cross-sectional HRTEM image of the 1Tʹ-MoTe_2_/Si Schottky junction in Fig. 3b obviously reveals the layer-by-layer structure along [001] direction for the MoTe_2_ layers with a thickness of ~34.3 nm on the Si substrate, yielding a sharp atomic heterostructure interface. Since the thickness of the monolayer MoTe_2_ is ~0.7 nm, the layer number is approximately determined to be ~49 layers. Due to the unique vdW growth manner, there are no obvious defects, including crystal deformation, stack faults, or dislocations at the interface, suggesting the formation of a high-quality junction interface. Significantly, the existence of an ultrathin natural oxidation layer (~4 nm) on the Si surface has an inappreciable effect on carrier transportation due to the direct tunneling behavior, which will even serve as a passivation layer to suppress carrier recombination and enhance device performance^1^. The corresponding elemental mapping results of the heterostructure also confirm the chemical element distributions in the 1Tʹ-MoTe_2_/Si Schottky junction.

Figure 3c plots the current-voltage (*I-V*) characteristics of the Gr/1Tʹ-MoTe_2_/Si Schottky junction photodetector under dark conditions and light illumination with various wavelengths. In the dark, the device shows a typical current rectification characteristic with a remarkable current ratio, suggesting the high quality of the Schottky junction. Notably, upon light illumination in a broad wavelength range (265 nm–10.6 μm), the generation of photon-excited carriers results in the right shift of *I-V* characteristic curves, giving rise to pronounced photovoltaic behaviors, which enable the device to operate in a self-powered mode without the need of extra power. We further characterized the photovoltaic photoresponse properties of the device over a wide spectral range, as shown in Fig. 3d. Notably, the photodetector demonstrates the robust response to ultrabroadband light from deep UV of 265 nm to LWIR of 10.6 μm with fast speed, giving rise to decent *I*_on_/*I*_off_ ratios of ~10^5^ for 265 nm (1.7 mW cm^−2^), ~10^6^ for 405 nm (32 mW cm^−2^), ~4 × 10^6^ for 650 nm (43 mW cm^−2^), ~5.6 × 10^6^ for 980 nm (44 mW cm^−2^), ~1.4 × 10^4^ for 1550 nm (23 mW cm^−2^), ~1.3 × 10^3^ for 2.2 μm (50 mW cm^−2^), ~6.6 × 10^2^ for 3.0 μm (50 mW cm^−2^), ~4.8 × 10^2^ for 4.6 μm (50 mW cm^−2^), and ~3.7 × 10^2^ for 10.6 μm (50 mW cm^−2^). The photovoltaic response spanning from deep UV to LWIR can be ascribed to the ultrawide broad absorption of 2D semimetallic 1Tʹ-MoTe_2_ layer. The working mechanism could be explained by the energy band diagram of the Schottky junction (Fig. 3e). Due to the difference in the work function of the multilayer MoTe_2_ layer and *n*-Si, a built-in electric field at the junction interface will be produced with a Schottky barrier of ~0.38 eV when the 1Tʹ-MoTe_2_ layer contacts with *n*-Si. Based on the electric field distribution simulation of the 1Tʹ-MoTe_2_/Si Schottky junction by COMSOL Multiphysics (Fig. 3f), a depletion layer is mainly distributed at the Si side with a built-in electric field direction from Si to 1Tʹ-MoTe_2_. Under UV-near infrared (NIR) light illumination with photon energy higher than the bandgap of Si, the photogenerated electron-hole pairs from both the 1Tʹ-MoTe_2_ and Si sides will be separated by the built-in electric field and then drift in the opposite direction toward each electrode, giving rise to the photocurrent. However, upon IR illumination with sufficient energy to overcome the Schottky barrier, the electrons would flow from the 1Tʹ-MoTe_2_ to the Si side with the thermionic emission mechanism, resulting in an obvious photocurrent in the NIR-MIR range. For the IR photon with energy lower than the Schottky barrier, the photo-excited carriers can transit through the barrier of the ultrathin insulator layer (~4 nm) based on a direct tunneling working mechanism, producing the pronounced photoresponse of up to 10.6 µm^1^.

**Fig. 4:**
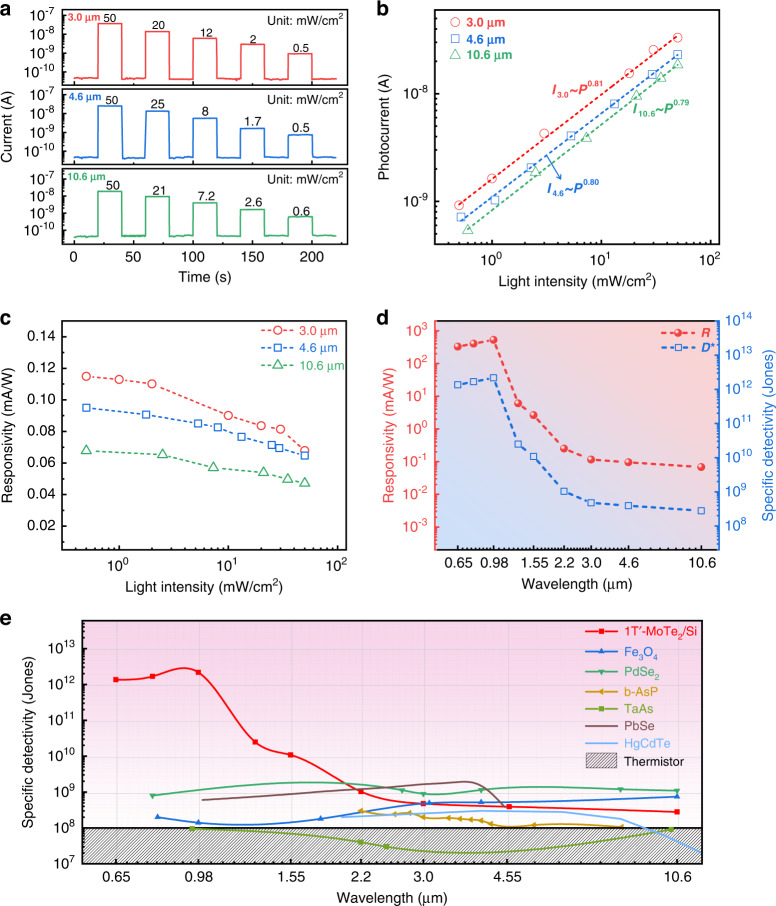
MIR photodetection performance of the photodetector. **a** Temporal photoresponses of the Gr/1Tʹ-MoTe_2_/Si Schottky junction device under light illumination of 3, 4.6, and 10.6 μm with various light intensities. **b** The fitting photocurrent versus light intensity at 3.0, 4.6, and 10.6 μm by the power law. **c** Responsivity at 3.0, 4.6, and 10.6 μm as a function of light intensity. **d** Responsivity and specific detectivity of the photodetector over a broad spectral range. **e** Comparison of the room temperature specific detectivity of the Gr/1Tʹ-MoTe_2_/Si Schottky junction device with other IR detectors

The MIR photodetection performance of the 1Tʹ-MoTe_2_/Si Schottky junction device was evaluated by investigating photoresponses of the device under MIR illumination of 3.0, 4.6, and 10.6 µm. Obviously, the photodetector shows prominent MIR responses when the lasers were periodically switched on and off, as shown in Fig. 4a. We believed that the photovoltaic effect could account for the pronounced photoresponse with sharp fall and rise edges rather than the thermal electric or bolometer effect^30^. In Fig. 4b, we fitted the light intensity-dependent photocurrents with a power law (*I*_ph_∼*AP*^θ^, where *I*_ph_ is the photocurrent, and *θ* determines the photoresponse to the power density), giving the power exponents of 0.81, 0.80, and 0.79 for 3.0, 4.6, and 10.6 μm, respectively, indicating the complex carrier recombination processes in the device^34,35^. Furthermore, the responsivity (*R*) and specific detectivity (D*) are evaluated by the formulas of

1 $$R = \frac{{I_{{\mathrm{ph}}}}}{{{\mathrm{PS}}}} = \frac{{I_{{\mathrm{light}}} - I_{{\mathrm{dark}}}}}{{{\mathrm{PS}}}}$$

2 $$D^ \ast = \frac{{\sqrt {A\Delta f} }}{{{\mathrm{NEP}}}}$$

where *I*_light_ and *I*_dark_, are the current under light illumination and in the dark, *P, S, A*, Δ*f*, and *NEP* are light intensity, effective irradiated area, device area, bandwidth, and noise equivalent power, respectively^36^. Figure 4c presents the calculated *R* under MIR illumination with various light intensities. *R* is found to decrease with increasing light intensity and reaches 0.115, 0.095, and 0.068 mA W^−1^ at zero bias under 3.0, 4.6, and 10.6 µm illumination (0.5 mW cm^−2^), respectively. The measured noise current density gives a value of 9.74 × 10^−14^ A Hz^−1/2^ at Δ*f* = 1 Hz (Fig. S13). Hence, the room temperature D* is determined to be 4.75/3.92/2.80 × 10^8^ Jones under 3.0/4.6/10.6 µm illumination. In addition, *R* and D* of the Schottky junction device are determined to be 526 mA W^−1^ and 2.17 × 10^12^ Jones under 980 nm illumination (Fig. S14), which are superior to that of photodetectors based on few-layer 2H-MoTe_2_ (24 mA W^−1^, 1.3 × 10^9^ Jones)^37^, MoTe_2_ p-n junction (4.8 mA W^−1^)^38^, MoTe_2_/Graphene (1.55 × 10^11^ Jones)^39^, the strain-engineered MoTe_2_ photodetector (0.5 A W^−1^)^40^, and 1T′-MoTe_2_ (96 mA W^−1^)^41^ operating in NIR region. The *R* and D* of the photodetector in a wide wavelength range reveal an ultrabroadband photoresponse spectrum range up to LWIR of 10.6 μm (Fig. 4d), which is among the widest range for MoTe_2_-based heterostructure photodetectors, such as 2H-MoTe_2_/Si (300–1800 nm)^42^, PdSe_2_/MoTe_2_ (365–980 nm)^43^, MoTe_2_/graphene (532–1064 nm)^39^, and MoTe_2_/MoS_2_ (550–1550 nm)^34^. It is expected that the use of other narrow bandgap semiconductors (such as HgCdTe, InGaAs, and Ge) as the substrates could effectively adjust the barrier heights of Schottky junctions, and make it possible to further improve the photodetection performance of the device with a detection range beyond 10.6 μm^44^. In addition, the photodetector has a large D* over 10^8^ Jones in the MIR region, as summarized in Fig. 4e. The obtained room temperature D* is not only superior to InSb (8.1 × 10^6^ Jones at 2.7 µm)^45^, T_d_-MoTe_2_ (9.1 × 10^6^ Jones at 10.6 µm)^24^, b-AsP (2.02/1.61/1.06 × 10^8^ Jones at 3.0/4.0/8.05 µm)^16^, HgCdTe (3 × 10^8^/6 × 10^7^ Jones at 3.0/10 µm)^46^, TaAs (9 × 10^7^ Jones at 10.29 µm)^47^, and the commercial thermistor bolometers (~10^8^ Jones), but also comparable to Fe_3_O_4_ (4.9/5.2/7.4 × 10^8^ Jones at 3.0/4.6/10.6 µm)^48^, PbSe (1.6 × 10^9^/3.8 × 10^8^ Jones at 3.0/4.5 µm)^49^, and PdSe_2_ (9.1 × 10^8^/1.2 × 10^9^/1.1 × 10^9^ Jones at 3.0/4.6/10.6 µm)^50^.

**Fig. 5:**
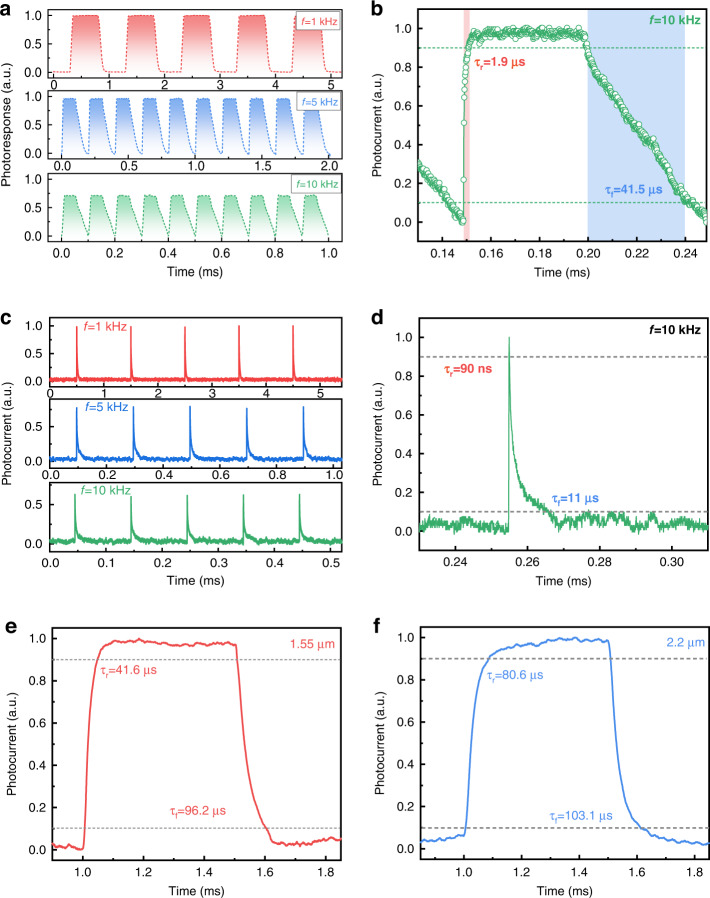
Response speed of the photodetector. **a** Photoresponse properties of the photodetector to 980 nm pulse signals with a duty ratio of 50%. **b** Rising and falling time at 10 kHz with a duty ratio of 50%. **c** Photoresponse properties of the device to 980 nm pulse signals with a duty ratio of 0.1%. **d** Rising and falling time at 10 kHz with a duty ratio of 0.1%. Response speeds of the device at **e** 1.55 and **f** 2.2 μm, respectively

Furthermore, the response speeds of the Schottky junction device under IR illumination are characterized. As shown in Fig. 5a, the photodetector exhibits stable and fast photoresponses to modulated laser diode illumination (λ = 980 nm) with switching frequencies of 1, 5, and 10 kHz at a duty ratio of 50%. A large 3-dB frequency (*f*_3dB_) of 10 kHz is achieved for the device, revealing its great capability of detecting fast-varying optical signals, even up to 50 kHz (Fig. S15). A short rising/falling time of 1.9/41.5 µs is obtained at *f*_3dB_ of 10 kHz (Fig. 5b). As a matter of fact, the Gr/1Tʹ-MoTe_2_/Si Schottky junction photodetector can follow ultrafast short-pulsed signals (duty ratio of 0.1%) at switching frequencies of 1, 5, and 10 kHz with good stability and distinguishability (Fig. 5c). Under a single pulse at 10 kHz with a pulse width of 100 ns, the photodetector features a fast rising/falling time of 90 ns/11 µs, which is among the fastest for TMD/Si photodetectors^35,51–58^. The fast response speed of the photodetector could be attributed to the following aspects: (i) The type-II Weyl semimetal 1T′-MoTe_2_ layers with high carrier mobility possess a unique orthorhombic lattice structure with broken inversion symmetry, ensuring the rapid carrier transportation and thus reducing carrier recombination^24^. (ii) The large built-in electric field in the device plays an important role in facilitating the fast separation and transportation of the photogenerated carriers^59^. (iii) The vertically stacked heterostructure with top transparent graphene contact would shorten the transit time of the carriers and reduce the carrier recombination induced by the grain boundaries in the 2D MoTe_2_ layer, leading to a fast response speed^42^. The above reasons corporately result in the short response time of the photodetector. Besides, the longer fall time could be attributed to the existence of some defects and traps in MoTe_2_ layers and junction interface. The photo-excited carriers trapped by the defects and traps will be slowly released by turning off the light, resulting in a relatively longer falling time^60^. Furthermore, the response speeds of 41.6/96.2 µs and 80.6/103.1 µs are achieved under 1.55 and 2.2 μm light illumination, respectively (Fig. 5e, f), which are probably due to the lower carrier concentration generated under IR light illumination with longer wavelength^61^. Benefiting from the fast response speed of the detector, we explored its application of a new-concept visual demonstration in IR optical communication. As shown in Fig. S16a, the target information of “MoTe2” entered into a computer was converted to American Standard Code for Information Interchange (ASCII) codes and then was sent to a signal generator to drive an infrared laser of 1550 nm (C-band). Subsequently, the conversion of the modulated pulsed IR signals to electrical signals by the Gr/1Tʹ-MoTe_2_/Si Schottky junction device, transmitted to a terminal computer *via* an oscilloscope, produced the resulting ASCII codes of “MoTe2” (Fig. S16b). The perfect square waves matched well with the original input information, suggesting the great potential of the device for IR optical communication.

**Fig. 6:**
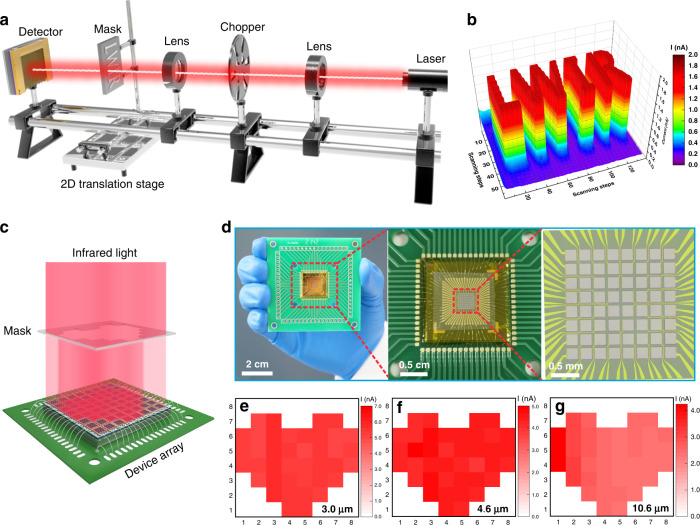
MIR imaging application of the photodetector. **a** A schematic of a single-pixel-based IR imaging system. **b** IR imaging of “LWIR” patterns under 10.6 μm at room temperature. **c** A schematic of IR imaging measurement based on devices array. **d** Photographs of an 8 × 8 1Tʹ-MoTe_2_/Si Schottky junction device array. The device area of each pixel cell is 200 × 200 μm^2^. **e**–**g** The imaging results of the “heart” pattern under 3.0, 4.6, and 10.6 μm, respectively

Given the superior IR detection capability of the photodetector, the room temperature IR imaging was further explored with the Gr/1Tʹ-MoTe_2_/Si Schottky junction device. Figure 6a demonstrates a schematic of the imaging measurement system with an individual Gr/1Tʹ-MoTe_2_/Si device as a sensing pixel, from which the IR light signal passed through a hollow LWIR-patterned mask fixed on a 2D translation stage. Subsequently, the position-dependent photocurrent of the device was recorded by the software-programmed computer and then transformed to a corresponding photocurrent mapping image of “LWIR” patterns under 10.6 μm in the absence of cryogenic cooling. As shown in Fig. 6b, the imaging result of clear “LWIR” patterns (134 × 59 steps) with a large current contrast ratio over 10 and sharp edges is acquired at room temperature. Furthermore, the large-scale uniform 2D MoTe_2_ layer enables the fabrication of an 8 × 8 1Tʹ-MoTe_2_/Si Schottky junction device array for IR imaging application (Fig. 6c, d). Notably, the uniform distributions of the dark current and the photocurrent under 10.6 μm with tiny fluctuation, according to the current mappings results in Fig. S17, reveal the satisfactory performance uniformity of the device array. Upon MIR illumination, the large difference between the currents from exposed pixels and unexposed counterparts results in a high-resolution heart-shaped image with large current ratios of 100, 68, and 51 for 3.0, 4.6, and 10.6 μm laser illumination at room temperature, respectively. Such excellent room temperature imaging capability with good homogeneity of the device array certainly confirms its great promise for MIR imaging applications. The wafer-scale growth of the 2D MoTe_2_ layer compatible with Si technology shows great potential for next-generation on-chip Si CMOS systems with low-power consumption and low-cost production.

## Discussion

In summary, we demonstrated the vdW growth of wafer-scale 2D MoTe_2_ layers with controllable phases of 2H (semiconductor) and 1Tʹ (semimetal) *via* a thermal-assisted tellurization method. Through in situ vdW growth of 1Tʹ-MoTe_2_ layers on a pre-patterned Si substrate, high-quality 1Tʹ-MoTe_2_/Si 2D/3D vdW Schottky junction was fabricated to achieve broadband IR detection. Thanks to the 2D MoTe_2_ layers with few interface defects via the vdW epitaxial growth mode, the wide absorption of the type-II Weyl semimetal 1Tʹ-MoTe_2_ layer, and the high-quality vertical junction with transparent graphene electrode, the Gr/1Tʹ-MoTe_2_/Si Schottky junction device has the capability of detecting an ultrawide light signal up to 10.6 μm at room temperature. This makes it one of the widest for 2D material-based photodetectors. Moreover, the device shows a large specific detectivity of 4.75–2.8 × 10^8^ Jones in the range of 3.0–10.6 µm with a fast response time. The wafer-scale growth of 2D MoTe_2_ layers also enable the assembly of a photodetector array to achieve an excellent room temperature MIR imaging capability. Our work demonstrates a novel design concept for the fabrication of high-performance, uncooled broad IR photodetectors based on 2D layered materials.

## Materials and methods

### Materials synthesis and characterization

Wafer-scale 2D MoTe_2_ layers were fabricated *via* a thermal-assisted tellurization route. In detail, Mo precursor layers were first defined on pre-cleaned substrates by a magnetron sputtering system. Then, the Mo-coated substrates were placed on a quartz boat filled with Te powder (99.99%) at the center of a horizontal tube furnace. The tube was evacuated and filled with a gas mixture of Ar (95%) and H_2_ (5%) at 50 sccm. Afterward, the Mo samples and Te powder were heated to 700 °C. During the heating process, the MoTe_2_ layers can be produced by the transportation of vaporized Te to the Mo samples. The prepared 2D MoTe_2_ layers were analyzed by XRD (RigakuSmart Lab), Raman spectrometry (Horiba HR800), AFM (Veeco Nanoscope V), a field-emission-gun scanning electron microscope (LEO 1530) attached with an EBSD detector (Oxford Instruments NordlysNano EBSD detector and AZtecHKL), a UV/Vis/NIR spectrometer (PERKIN ELMER), STEM (JEOL JEM-ARM300F), and XPS (Thermo ESCALAB 250).

### Theoretical simulation

Our first-principles calculations based on density functional theory were performed by the Vienna Ab initio Simulation Package (VASP). For the exchange-correlation functions, we employed the generalized gradient approximation (PBE-GGA) with projector augmented wave (PAW) potentials^62–65^. We adopted the zero damping DFT-D3 method of Grimme to describe van der Waals interactions^66^. The kinetic energy cutoff for the plane wave basis was set to 300 eV, and 8 × 8 × 2 (4 × 8 × 2) and 8 × 8 (4 × 8) meshes of *k*-space integration were used in the Brillouin zones of bulk and finite layer for 2H (1Tʹ) MoTe_2_, respectively. The finite-layer structures were simulated by a periodic slab model with a vacuum thickness of 15 Å to avoid periodic slab interactions. All atoms were allowed to relax until all residual force components were less than 0.01 eV.

The spatial distributions of the electric field intensity within the 1Tʹ-MoTe_2_/Si Schottky junction were investigated by the radio frequency (RF) module of COMSOL Multiphysics through an exact full-field electromagnetic calculation in the frequency domain based on Maxwell’s equations.

### Device fabrication and characterization

A SiO_2_ window (2 mm × 2 mm) was first defined on a SiO_2_ (300 nm)/Si (*n*-type, resistivity of 1–10 Ω cm) wafer through a wet etching process. Then, the 1Tʹ-MoTe_2_ layer was directly deposited on a pre-patterned SiO_2_/Si wafer to construct a 1Tʹ-MoTe_2_/Si Schottky junction device. Afterward, the Au (50 nm) and In-Ga alloy electrodes were defined on the MoTe_2_ layer and the back side of the Si wafer, respectively. Then a monolayer graphene film on the top surface of MoTe_2_ was chosen as the transparent electrode. The electrical and optoelectrical properties of the devices were investigated by a Keithley 4200-SCS (Tektronix), an oscilloscope (DPO2012B, Tektronix), and light sources with various wavelengths. The noise current of the device was measured by a semiconductor parameter analyzer system (Fs-pro, Primarius). Imaging measurements were performed with a lab-built imaging system. The hollow mask with “LWIR” patterns was mounted on a 2D motorized positioning system, programmed to move in a plane with each step of 0.5 mm. The device was located behind the mask. The position-dependent current of the device was detected by a lock-in amplifier when the mask moved.

## Supplementary information

Supplementary Information for Phase-Controlled van der Waals Growth of Wafer-Scale 2D MoTe2 Layers for Integrated High-Sensitivity Broadband Infrared Photodetection

## Data Availability

The data that support the findings of this study are available from the corresponding author upon reasonable request.
