# Supplementary material for: Phase-controlled van der Waals growth of wafer-scale 2D MoTe2 layers for integrated high-sensitivity broadband infrared photodetection
Source: Light Sci Appl. 2023 Jan 2;12:5. doi: 10.1038/s41377-022-01047-5 (PMC9806107; doi:10.1038/s41377-022-01047-5)
Supplement: Supplementary file 1 — Supplementary Information for Phase-Controlled van der Waals Growth of Wafer-Scale 2D MoTe2 Layers for Integrated High-Sensitivity Broadband Infrared Photodetection [file 41377_2022_1047_MOESM1_ESM.docx]

**Supplementary Information for**

Phase-Controlled van der Waals Growth of Wafer-Scale 2D MoTe_2_ Layers for Integrated High-Sensitivity Broadband Infrared Photodetection

Di Wu^1^, Chenguang Guo^1^, Longhui Zeng^2*^, Xiaoyan Ren^1^, Zhifeng Shi^1^, Long Wen^3^, Qin Chen^3^, Meng Zhang^4^, Xin Jian Li^1*^, Chong-Xin Shan^1^, and Jiansheng Jie^4*^

^1^School of Physics and Microelectronics, Key Laboratory of Material Physics Ministry of Education, Zhengzhou University, Zhengzhou, Henan 450052, China

^2^Department of Electrical and Computer Engineering, University of California San Diego, La Jolla, CA 92093, USA

^3^Institute of Nanophotonics, Jinan University, Guangzhou, Guangdong 511443, China

^4^Institute of Functional Nano and Soft Materials (FUNSOM), Jiangsu Key Laboratory for Carbon-Based Functional Materials and Devices, Soochow University, Suzhou, Jiangsu 215123, China

Correspondence authors:

Xinjian Li

Email: lixj@zzu.edu.cn, Tel: +86-371-67766629

Longhui Zeng

Email: lhzeng.hfut@gmail.com, Tel: +1-858-2147102

Jiansheng Jie

Email: jsjie@suda.edu.cn, Tel: +86-512-65881265


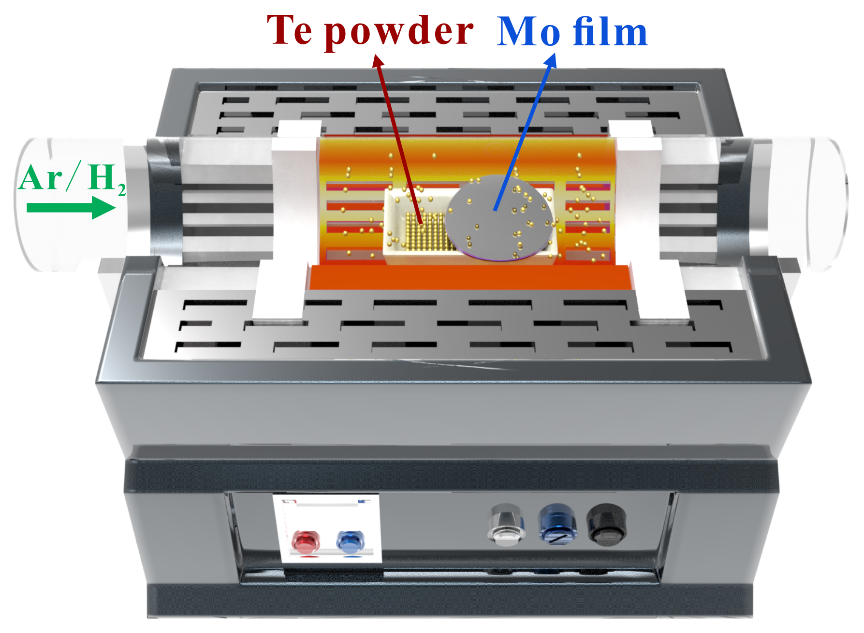


**Fig. S1** Schematic illustration of the synthesis of wafer-scale 2D MoTe_2_ layers.


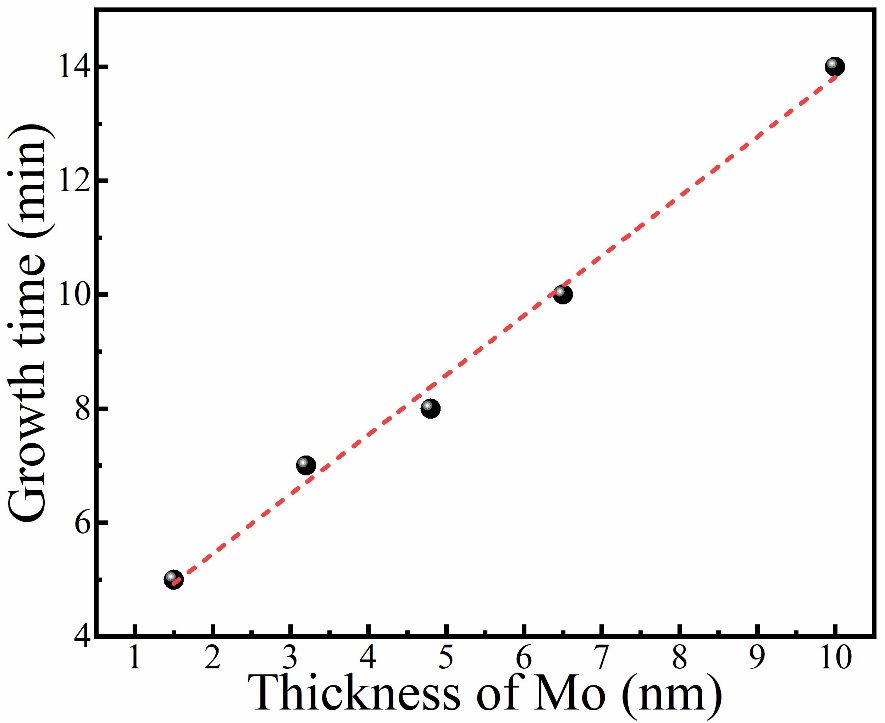


**Fig. S2** The thickness of the precursor Mo metal layer as a function of the growth time for 1T′-MoTe_2_.





**Fig. S3** The XRD patterns of 2D 1Tʹ- and 2H-MoTe_2_ layers.


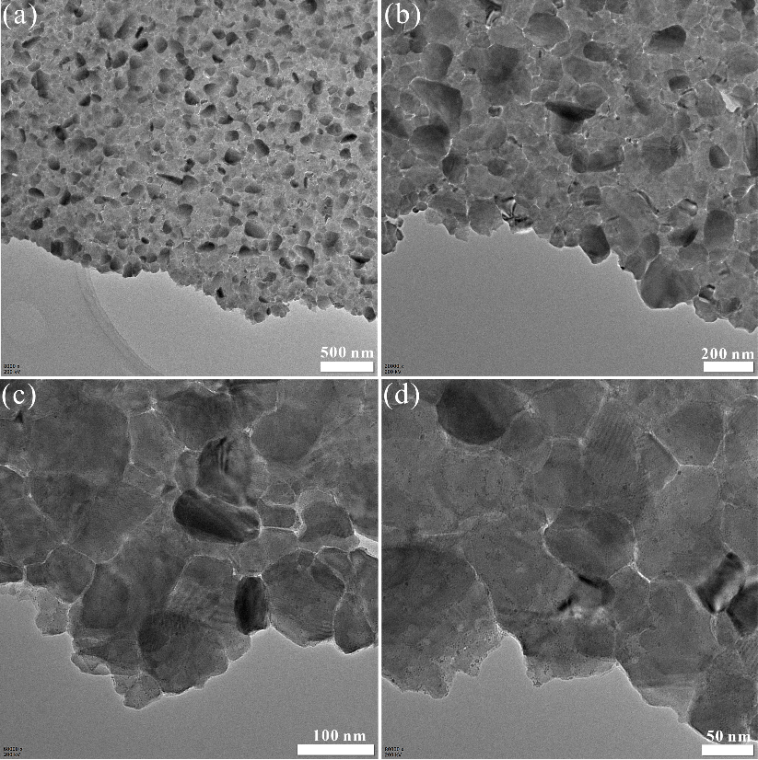


**Fig. S4** TEM images of 2D 1Tʹ-MoTe_2_ layers with different magnifications.


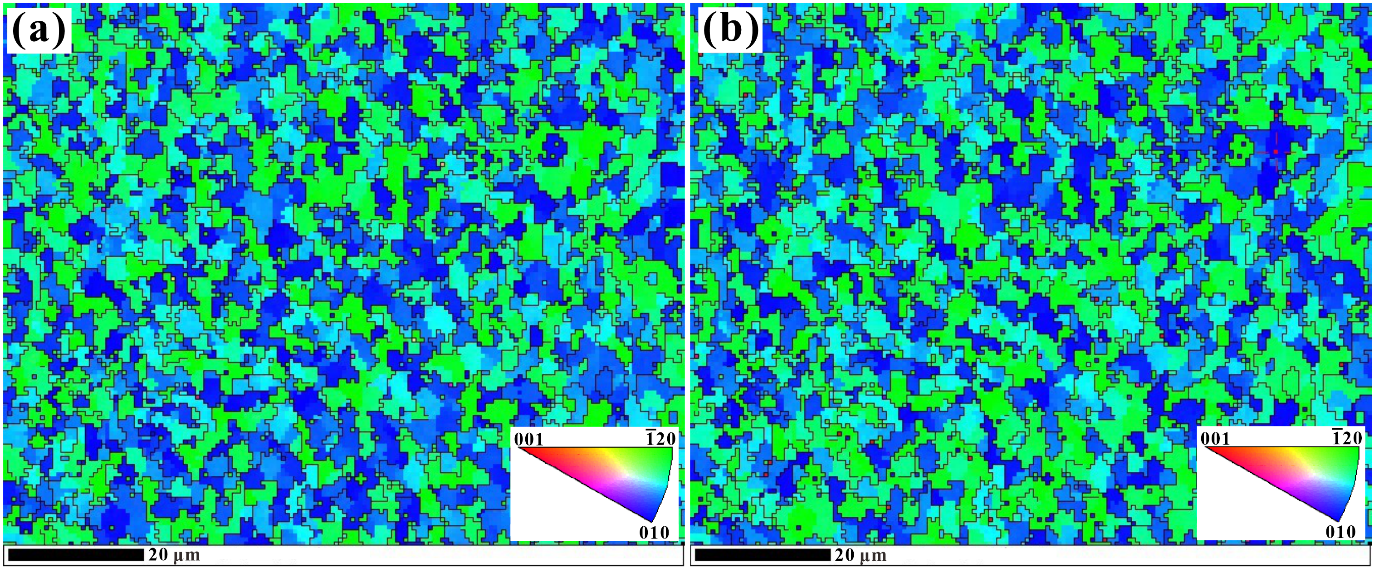


**Fig. S5** The inverse pole figure maps of the 1Tʹ-MoTe_2_ layer along the **a** *x*- and **b** *y*-axes.


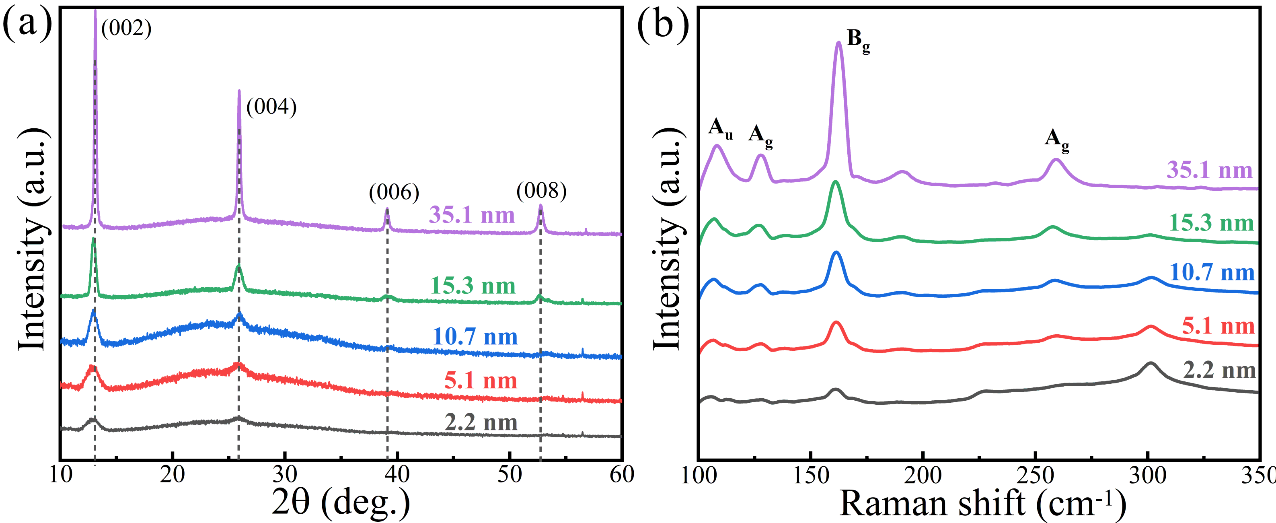


**Fig. S6** **a** XRD patterns and **b** Raman spectra of 1Tʹ-MoTe_2_ layers with different thicknesses.





**Fig. S7** The energy difference between the Fermi level (*E*_F_) and *E*_VBM_ extracted by UPS analysis.





**Fig. S8** The resistivity and mobility of 2H- and 1Tʹ-MoTe_2_ layers with different thicknesses.





**Fig. S9** Calculated electronic band structures of the 2D 2H-MoTe_2_ with different layer numbers.


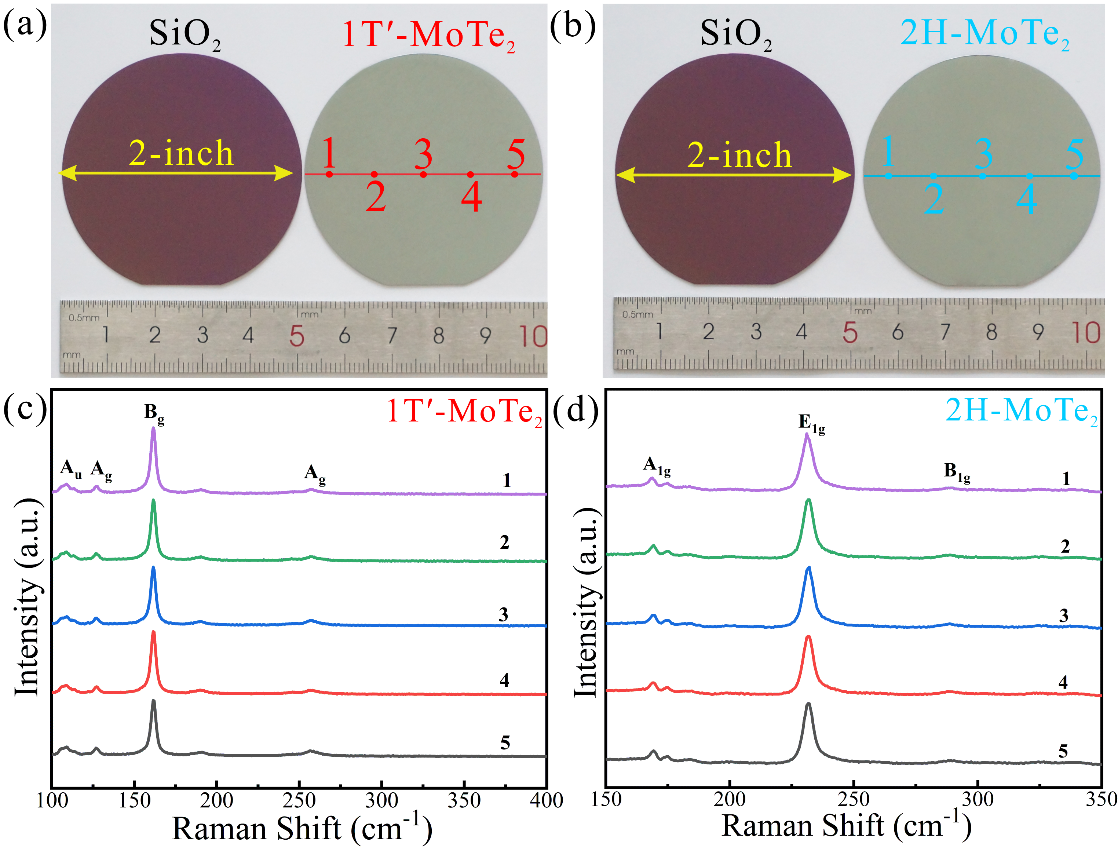


**Fig. S10** The wafer-scale **a** 1Tʹ- and **b** 2H-MoTe_2_ layers synthesized on a 2-inch SiO_2_/Si wafer. Raman spectra of the **c** 1Tʹ- and **d** 2H-MoTe_2_ layers.


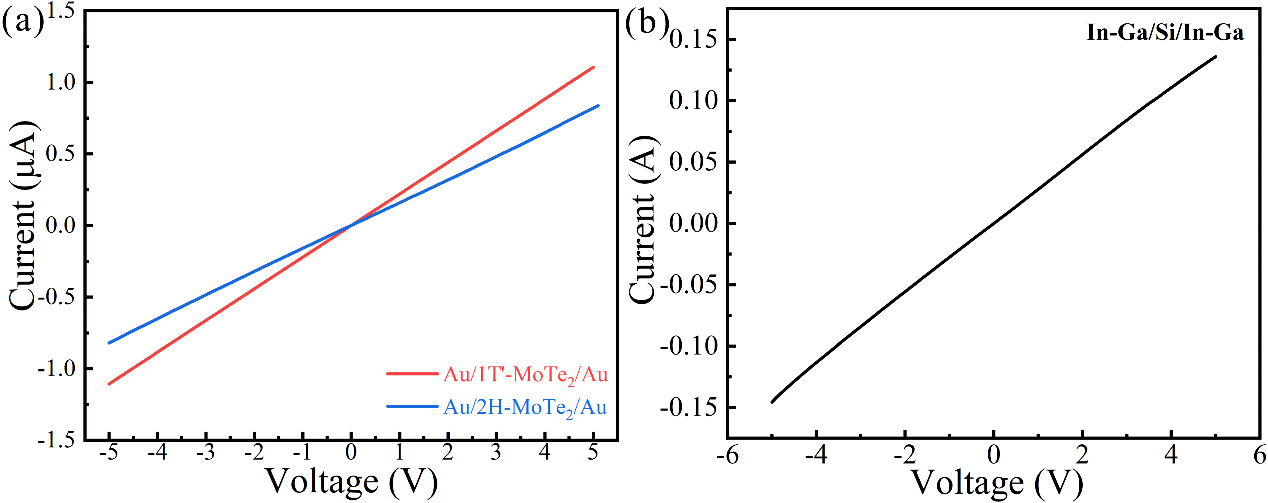


**Fig. S11** *I-V* curves of **a** Au/MoTe_2_/Au and **b** In-Ga/Si/In-Ga structures measured in the dark.


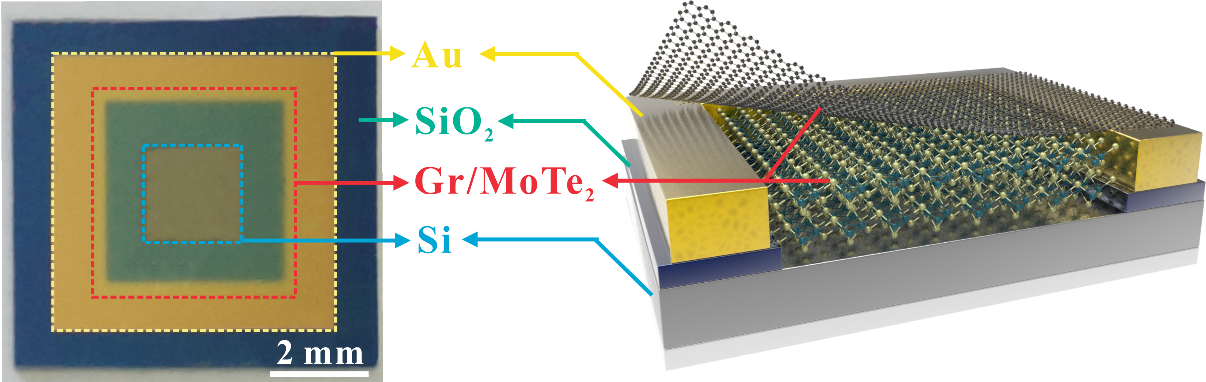


**Fig. S12** Photograph (left) and schematic illustration (right) of the Gr/1Tʹ-MoTe_2_/Si vertical Schottky junction device.





**Fig. S13** The measured current noise power spectrum of the Gr/1Tʹ-MoTe_2_/Si Schottky junction device at zero bias.


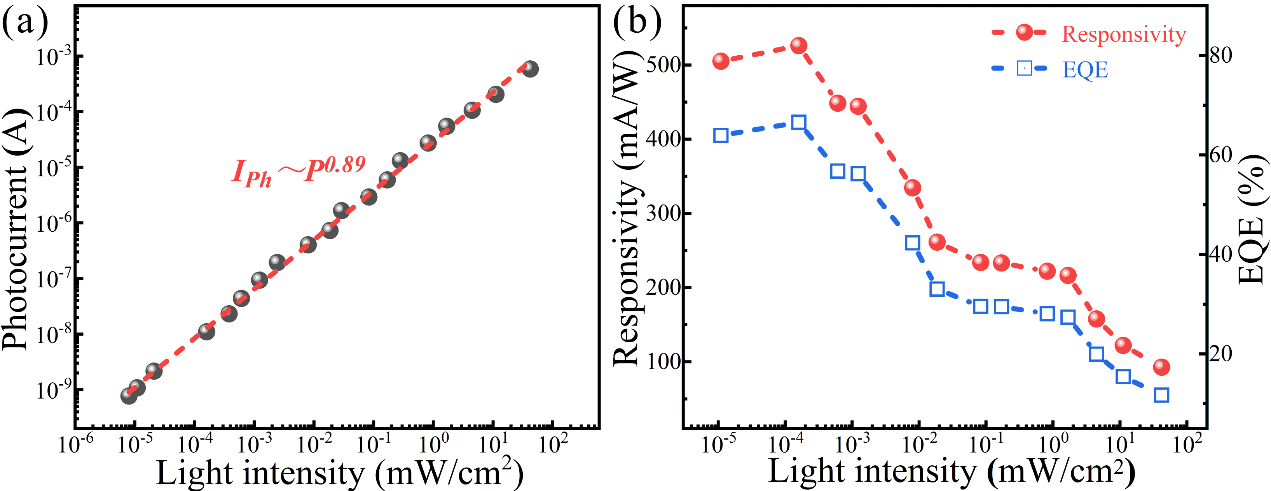


**Fig. S14** **a** Dependence of photocurrent on light intensity under 980 nm illumination. **b** Calculated *R* and *EQE* of the device at 980 nm as a function of light intensity.





**Fig. S15** Relative balance of [(*I*_max_ - *I*_min_)/*I*_max_] as a function of the frequency.


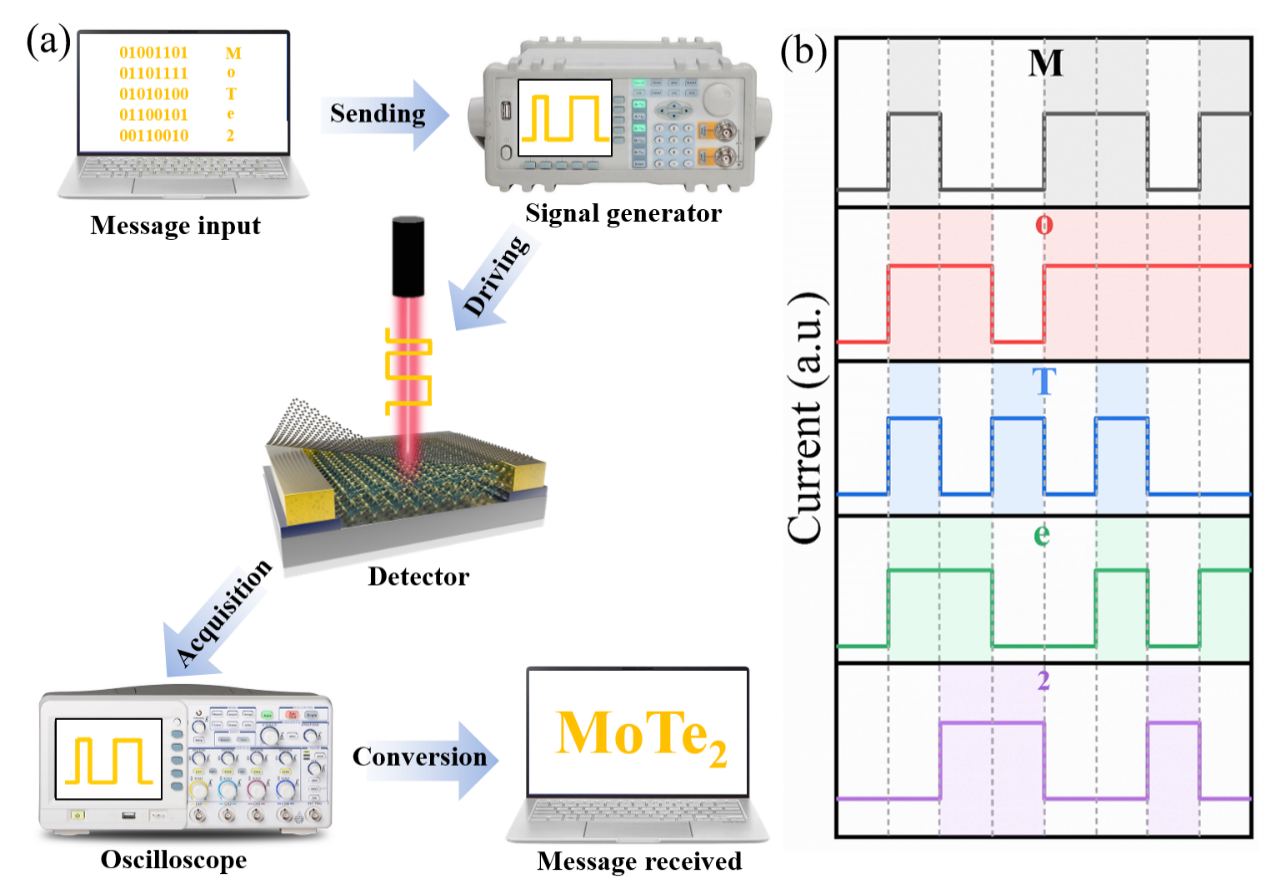


**Fig. S16** **a** Schematic diagram of the optical communication system. **b** The received signals of ASCII codes of “MoTe2” at 1.55 μm.


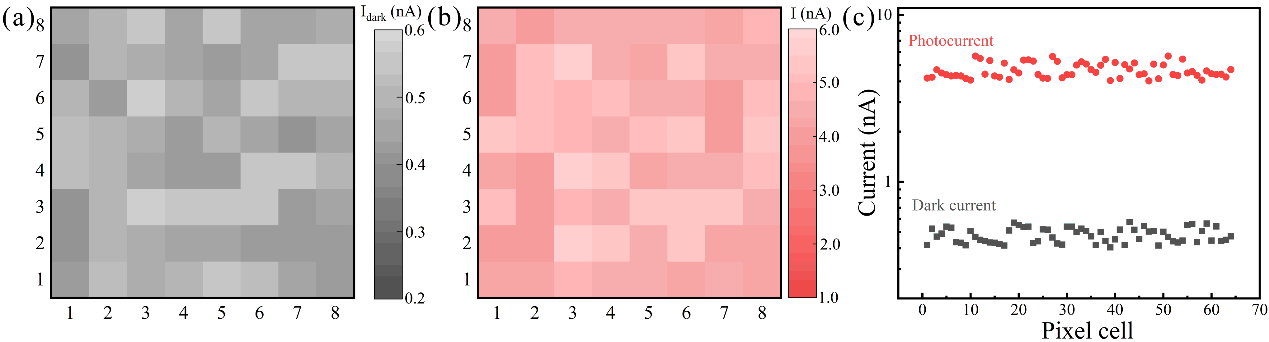


**Fig. S17** **a** Dark current mapping and **b** photocurrent mapping under 10.6 μm illumination of the 8×8 Gr/1Tʹ-MoTe_2_/Si Schottky junction device array. **c** Numerical statistics of the dark current and photocurrent.
